# Meeting medical emergency response criteria for hypertension is not associated with an increased likelihood of in-hospital mortality in a tertiary referral center^☆^

**DOI:** 10.1016/j.resplu.2024.100679

**Published:** 2024-06-05

**Authors:** Jin Nuo Joan Tsang, Stephen Bacchi, Christopher D. Ovenden, Rudy Goh, Joshua G. Kovoor, Aashray K. Gupta, Yong Min Le, Antoinette Lam, Brandon Stretton, Minh-Son To, Richard Woodman, Arduino A Mangoni, James Malycha

**Affiliations:** a College of Medicine and Public Health, Flinders University, Bedford Park SA 5042, Australia; b University of Adelaide, Adelaide SA 5005, Australia; c Royal Adelaide Hospital, Central Adelaide Local Health Network, Adelaide SA 5000, Australia; d Queen Elizabeth Hospital, Central Adelaide Local Health Network, Woodville SA 5011, Australia; e Gold Coast University Hospital, Southport QLD 4215, Australia; f Flinders Medical Centre, Southern Adelaide Local Health Network, Bedford Park SA 5042, Australia

**Keywords:** Hypertension, Hospital medicine, Medical emergency response, Vital signs

## Abstract

**Backgrounds:**

Rapid response team or medical emergency team (MET) calls are typically activated by significant alterations of vital signs in inpatients. However, the clinical significance of a specific criterion, blood pressure elevations, is uncertain.

**Objectives:**

The aim of this study was to evaluate the likelihood ratios associated with MET-activating vital signs, particularly in-patient hypertension, for predicting in-hospital mortality among general medicine inpatients who met MET criteria at any point during admission in a South Australian metropolitan teaching hospital.

**Results:**

Among the 15,734 admissions over a two-year period, 4282 (27.2%) met any MET criteria, with a positive likelihood ratio of 3.05 (95% CI 2.93 to 3.18) for in-hospital mortality. Individual MET criteria were significantly associated with in-hospital mortality, with the highest positive likelihood ratio for respiratory rate ≤ 7 breaths per minute (9.83, 95% CI 6.90 to 13.62), barring systolic pressure ≥ 200 mmHg (LR + 1.26, 95% CI 0.86 to 1.69).

**Conclusions:**

Our results show that meeting the MET criteria for hypertension, unlike other criteria, was not significant associated with in-hospital mortality. This observation warrants further research in other patient cohorts to determine whether blood pressure elevations should be routinely included in MET criteria.

## Introduction

Accurate prognostication during medical emergency team (MET) calls is vital for minimizing unnecessary interventions, but estimating this can be challenging. Previous studies have focused on the odds ratio of individual vital signs for predicting specific outcomes in patient with MET calls,^1^ and the influence on prognosis of meeting MET criteria in the emergency department.^2^ However, little attention has been paid for one such criterion, elevations in blood pressure.

MET calls for hypertension are typically triggered by systolic blood pressures > 200 mmHg^3^. Such values would meet the criteria for a hypertensive urgency in the absence of signs and symptoms of acute end organ damage, or hypertensive emergency in the presence of end-organ damage.^4^ The management of acute increases in blood pressure varies widely.^5^ While a Cochrane review has reported the lack of evidence demonstrating the association with morbidity or morality improvements with the use of antihypertensive treatment,^6^ international guidelines highlight the need for blood pressure reduction to avoid progressive organ failure.^4^ However, there is evidence that aggressive blood pressure lowering may cause hypotension, tachycardia, and higher rates of subsequent acute kidney injury and myocardial injury.^7^

Given the current uncertainties on the clinical significance of blood pressure elevations in hospitalized patients, we explored the association between in-hospital hypertension and clinical outcomes by evaluating the likelihood ratios associated with MET-activating vital signs and in-hospital mortality.

## Methods

We conducted a retrospective cohort study of non-selected consecutive patients admitted to general medicine wards at the Royal Adelaide Hospital, a 800-bed tertiary referral center and teaching hospital, over a two-year period between March 1st 2020 and March 1st 2022. Cases of primary severe hypertension and organ damage which would necessitate direct transfer to the high dependence unit or intensive care unit were excluded. In this hospital, MET activation is based on alterations of specific vital signs (Table 1) and results in immediate review by a medical registrar, MET resident medical officers and interns, and an intensive care nurse.

**Table 1:** MET activation criteria.

Parameter	Lower MET threshold	Upper MET threshold
Systolic blood pressure (mmHg)	≤89	≥200
Heart rate (beats per minute)	≤39	≥140
Respiratory rate (breaths per minute)	≤7	≥31
Oxygen saturation (%)	≤88	N/A
Supplemental oxygen flow rate (L/minute)	N/A	≥8
Sedation score	N/A	≥3

Data extracted from administrative and departmental databases and electronic medical records capturing clinical information throughout hospitalization included demographic factors (age and sex), vital signs, and in-hospital mortality. Individuals who met the MET activation criteria at any stage were identified. Missing input data were replaced with median imputation. Descriptive statistics were employed. Calculation of likelihood ratios (LR) and likelihood ratio 95% confidence intervals (95% CI) was performed using bootstrapping method.^8^ The post-test probability of an outcome can be calculated, assuming the pre-test probability can be estimated.^9^ Data analysis was performed using Python and R (version 1.1.456). The project received approval from Central Adelaide Local Health Network (CALHN) Human Research Ethics Committee (reference number: 11643). Waiver of consent has been granted for access to all electronic and hard medical records.

## Results

The mean age of the 15,734 individuals admitted during the observation period was 69.6 ± 18.7 years, and 7,715 (49%) were female. A total of 887 patients died in hospital (5.6%) and 4,282 (27.2%) met the MET criteria during admission. Meeting the MET criteria conferred a positive LR of 3.05 (95% CI 2.93 to 3.18) for in-hospital mortality, with respiratory rate ≤ 7 breaths per minute (LR + 9.83, 95% CI 6.90 to 13.62) showing the strongest association, followed by heart rate ≤ 39 beats/min (LR + 8.13, 95% CI 6.26 to 10.32). Notably, of all MET criteria, a systolic blood pressure ≥ 200 mmHg was the only MET criterion with a non-significant LR (LR + 1.26, 95% CI 0.86 to 1.69; Table 2). A total of 385 (2.5%) patients met the MET criteria for hypertension.

**Table 2:** Likelihood ratios of demographics and individual vital sign criteria for the prediction of in-hospital mortality.

Vital sign	Number of cases (n)	Positive LR (95%CI)	Negative LR (95%CI)
Respiratory rate ≤ 7 breaths per minute	100	9.83 (6.9–13.62)	0.96 (0.95–0.97)
Heart rate ≤ 39 beats per minute	205	8.13 (6.25–10.32)	0.93 (0.92–0.95)
Sedation score ≥ 3	249	7.08 (5.66–8.72)	0.93 (0.91–0.94)
Supplemental oxygen flow rate ≥ 8 L/minute	938	6.66 (6.02–7.35)	0.73 (0.71–0.76)
Respiratory rate ≥ 31 breaths per minute	1,324	5.45 (4.99–5.96)	0.68 (0.65–0.71)
Oxygen saturations ≤ 88%	2068	4.27 (3.99–4.56)	0.59 (0.56–0.62)
Heart rate ≥ 140 beats per minute	489	4.14 (3.45–4.95)	0.91 (0.9–0.93)
Systolic blood pressure ≤ 89 mmHg	1,658	3.7 (3.36–4.03)	0.73 (0.7–0.76)
Systolic blood pressure ≥ 200 mmHg	385	1.26 (0.86–1.69)	0.99 (0.98–1.0)
Meeting any vital sign criteria for medical emergency response activation	4,282	3.05 (2.93–3.18)	0.34 (0.31–0.37)
Age ≥ 80 years	5,698	1.69 (1.61–1.78)	0.63 (0.59–0.67)
Age ≥ 65 years	10,426	1.33 (1.3–1.36)	0.39 (0.33–0.44)
Female sex	7,715	0.86 (0.81–0.92)	1.13 (1.08–1.19)

Legend: LR, Likelihood ratio; CI, confidence interval.

## Discussion

In our study, meeting the criteria for MET activation was generally associated with a significantly increased likelihood of in-hospital mortality, particularly with low respiratory rate and low heart rate. Systolic blood pressure ≤ 89 mmHg also showed a positive association with mortality. This may be explained by the possibility of hypotension precipitating to septic shock, a life-threatening condition, in the setting of sepsis.^10^ However, meeting the MET criteria for systolic blood pressure ≥ 200 mmHg was not associated with an increased likelihood of in-hospital mortality, suggesting the presence of significance prognostic difference across MET criteria used in hospital practice.

Currently, there are no clear guidelines for treating inpatient hypertension, particularly in the absence of signs or symptoms of acute target organ damage.^11^ Situational factors such as pain, nausea, fever, anxiety, stress, erratic intake of existing antihypertensive agents, exposure of new drugs and white coat syndrome can transiently elevate blood pressure during hospitalization.^11^ However, it is unknown whether these responses are adaptive or harmful, adding to the complexity in predicting outcomes related to increased blood pressure.^12^ The non-significant positive likelihood ratio of systolic blood pressure ≥ 200 mmHg and in-hospital mortality suggests that a more conservative approach in managing inpatient hypertension may be reasonable except in high-risk patient groups such as acute ischemic stroke. This hypothesis is supported by previous research showing non-significant differences in adverse events between treated and non-treated hypertensive patients without acute target organ damage, while rapid reduction of blood pressure can increase risk of inadequate tissue perfusion and organ ischemia.^13^ This information is important in aiding decision making given that patients are frequently and inconsistently managed with as-needed antihypertensive medications.^14^ The results also support previous research that has identified a significant proportion of patients in general wards in Australian hospitals meeting MET criteria during their admissions.^15^ Various reasons can delay MET activation, such as lack of confidence and a fear of receiving criticism. Failure of MET activation may result in worsened outcomes including increasing length of stay.^16^.

The finding of associations between certain predefined MET criteria and mortality offers valuable insights into potential MET modifications for individuals in certain carefully considered circumstances. While a study of 430 patients suggested that such modifications have limited effect on outcomes, other retrospective studies have reported that modifications may reduce the potential efficacy of multi-tiered response systems.^17^ Notably, the lack of association between meeting MET criteria for hypertension and the likelihood of in-hospital mortality may inform modification placement.

While our study provides valuable insights, there are some limitations. Noting that this study was conducted at a single centre and limited to the general medicine population, further studies examining these factors in different hospitals and cohorts (eg surgical patients), and also considering the potential impact of several clinical and demographic characteristics, would improve external generalisability. Given the retrospective nature of the study, prospective studies may be beneficial which can have better control over exposure factors and potential confounders. Another limitation includes the lack of information regarding how blood pressure was measured, which may have introduced additional variability, and the duration of the reported elevations in systolic blood pressure. It is also unclear whether meeting the MET criteria for hypertension prompted the initiation of antihypertensives and for how long. The association is not adjusted for the number of MET calls that an individual had received. Future studies may consider examining alternative thresholds for MET activation particularly with respect to hypertension in asymptomatic patients without evidence of acute target organ damage. Further research could benefit from utilizing aggregate warning scores to investigate the significance of simultaneous abnormal parameters that may warrant MET calls. If the provision of this information is found to be beneficial, future research may seek to examine other areas including the likelihood ratios associated with outcomes following intensive care unit admission, intubation, and cardiopulmonary resuscitation.

## Conclusion

The majority of MET-activating vital signs were significantly associated with an increased likelihood of in-hospital mortality apart from inpatient systolic blood pressure ≥ 200 mmHg. Further studies may seek to investigate alternative MER activation criteria thresholds for hypertension and their effects on clinical and patient outcomes.

## Statement of financial support

This research was not financially funded.

## CRediT authorship contribution statement

**Jin Nuo Joan Tsang:** Writing – review & editing, Writing – original draft, Methodology, Data curation, Conceptualization. **Stephen Bacchi:** Writing – review & editing, Writing – original draft, Supervision, Methodology, Formal analysis, Data curation, Conceptualization. **Christopher D. Ovenden:** Writing – review & editing, Methodology, Data curation. **Rudy Goh:** Writing – review & editing, Methodology, Data curation. **Joshua G. Kovoor:** Writing – review & editing, Writing – original draft, Methodology, Data curation. **Aashray K. Gupta:** Writing – review & editing, Writing – original draft, Methodology, Data curation. **Yong Min Le:** Writing – review & editing, Methodology, Data curation. **Antoinette Lam:** Writing – review & editing, Methodology, Data curation. **Brandon Stretton:** Writing – review & editing, Methodology, Data curation. **Minh-Son To:** Writing – review & editing, Methodology, Data curation, Writing – review & editing, Methodology, Data curation. **Richard Woodman:** Writing – review & editing, Data curation. **Arduino A Mangoni:** Writing – review & editing, Methodology, Data curation, Conceptualization, Writing – review & editing, Methodology, Data curation, Conceptualization. **James Malycha:** Writing – review & editing, Methodology, Data curation.

## Declaration of competing interest

The authors declare that they have no known competing financial interests or personal relationships that could have appeared to influence the work reported in this paper.
